# Healthcare Systems across Europe and the US: The Managed Entry Agreements Experience

**DOI:** 10.3390/healthcare11030447

**Published:** 2023-02-03

**Authors:** Michele Ciulla, Lisa Marinelli, Giuseppe Di Biase, Ivana Cacciatore, Fiorenzo Santoleri, Alberto Costantini, Marilisa Pia Dimmito, Antonio Di Stefano

**Affiliations:** 1 Department of Pharmacy, University “G. d’Annunzio” of Chieti–Pescara, 66100 Chieti, Italy; 2 Pharmacy of Spirito Santo Hospital, 65124 Pescara, Italy

**Keywords:** drug pricing, health policies, managed entry agreements, pharmaceutical market, pharmaceutical risk sharing

## Abstract

This systematic study aims at analyzing the differences between the approach of the European healthcare systems to the pharmaceutical market and the American one. This paper highlights the opportunities and the limitations given by the application of managed entry agreements (MEAs) in European countries as opposed to the American market, which does not regulate pharmaceutical prices. Data were collected from the Organisation for Economic Co-operation and Development (OECD), the European Medicines Agency, and the national healthcare agencies of US and European countries. A literature review was undertaken in PubMed, Scopus, MEDLINE, and Google for a period ten years (2010–2019). The period 2020–2021 was considered to compare health expenditure before and after the SARS-CoV-2 pandemic. Scarce information from national agencies has been given in terms of MEAs related to the COVID-19 pandemic. The comparison between the United States approach and the European one shows the importance of a market access regulation to reduce the cost of therapies, increasing the efficiency of national healthcare systems and the advantages in terms of quality and accessibility to the final users: patients. Nevertheless, it seems that the golden age of MEAs for Europe was during the examined period. Except for Italy, countries will move to other forms of reimbursements to obtain higher benefits, reducing the costs of an inefficient implementation and outcomes in the medium term.

## 1. Introduction

**Figure 1:**
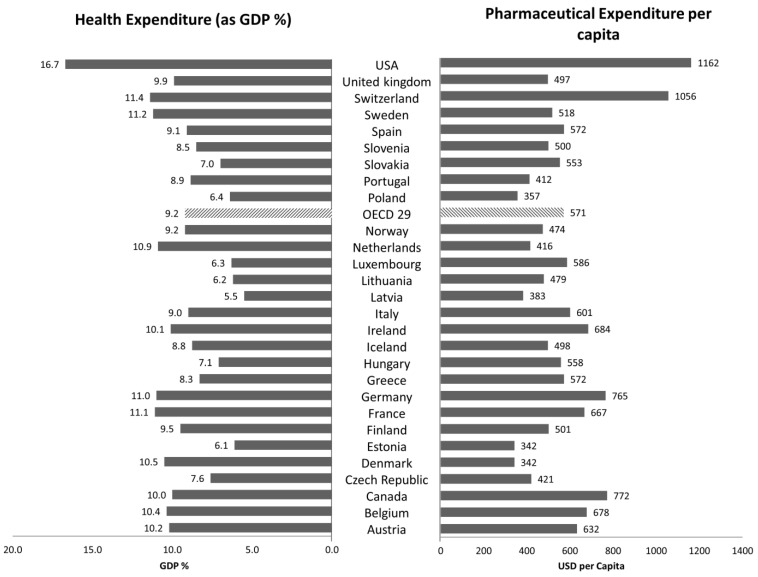
Health Expenditure as % of GDP and Pharmaceutical Expenditure per capita (in USD) of 29 OECD countries in 2015 [2].

Health does not have a price, but it has some costs. Healthcare systems take a fundamental role in countries to guarantee equal access to basic and advanced services for the entire population, improving welfare and the general quality of life. Evidence can be found by analyzing the healthcare expenditure in the percentage of national gross domestic product (GDP) for Organisation for Economic Co-operation and Development (OECD) countries. In 2015, the central year of the examined period (2010–2019), OECD countries spent 9% of GDP on healthcare on average, with the United States (US) almost doubling this value, the highest rate among industrialized nations (Figure 1) [1].

From the median of the examined period till the onset of the COVID-19 pandemic (2019), for OECD countries the average of health expenditure as % of GDP decreased from 9.2% to 8.7%, whereas for the US the same parameter was constant in both years at 16.7% [2].

**Figure 2:**
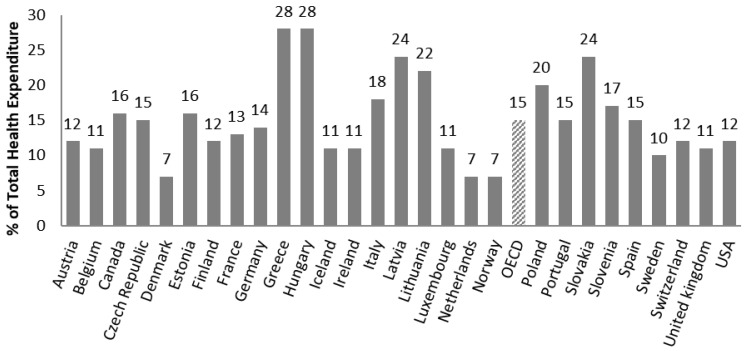
Pharmaceutical Spending as % of total Health Expenditure in 2019 (pharmaceuticals in hospitals are not considered) [2].

Since pharmaceuticals play a vital role within the panel of expenses in healthcare systems, regulation has a fundamental importance to guarantee access to new and effective medicines for patients. A regulation is often able to limit the healthcare budgets and provide the right incentives to manufacturers to develop new generations of drugs. Among OECD countries, the spending on retail pharmaceuticals represents a large part of healthcare costs. It is ranked second after inpatient and outpatient care. In 2019, it accounted for more than a sixth (15%) of health expenditure on average without considering the spending on pharmaceuticals in hospitals (Figure 2). In the past decade, the pharmaceutical market has increased at a slower pace than before, due to patent expiries of several blockbuster drugs and the economic crisis, which also influenced the national health cost-containment policies [2].

In 2015, countries spent an average of more than USD 550 per person on retail pharmaceuticals (Figure 1). The US showed an expenditure per capita more than twice higher than the average of OECD countries, and this high level can be ascribed to the different approach to health policies which will be analyzed later in this paper.

In 2019, the US spent USD 1277 per capita, with an increase of more than USD 100 compared to 2015, whereas the other OECD countries kept the pharmaceutical expenditure per capita constant [2]. The 2020–2021 period is examined further in the following section.

The increasing demand for medicines and the introduction of new therapies into the market are the main drivers of pharmaceutical spending growth. New and innovative drugs showed high prices for a single therapy, establishing an important barrier to patient access. Moreover, the increase in population aging along with the prevalence of many chronic diseases (cancer, diabetes, cholesterol-related pathologies, neurodegenerative diseases) pushed several countries to cost-containment policies in the pharmaceutical market in order to influence and reduce the spending trends. At the same time, they worked to increase the efficiency of their healthcare systems [3]. European policy-makers recognized high drug prices as the main challenge to overcome to provide access to new therapies, remaining within the limits of the national health budget. A range of policy measures has been applied to reduce pharmaceutical costs [4]. Among the possible restrictive actions, several countries shifted part of the burden of pharmaceutical spending to private payers. Another strategy was the implementation of measures to increase mandatory rebates on pharmaceutical companies, giving a predominant role to health technology assessment (HTA) in the reimbursement and/or pricing process [5]. Moreover, many European nations introduced the use of managed entry agreements (MEAs) as important tools to evaluate the clinical and cost-effectiveness of new drugs. Simplistically, MEAs are arrangements between the manufacturer and the payers which enable coverage/reimbursement of new therapies under defined conditions. These arrangements can use a variety of mechanisms to address uncertainty about new technologies and make a decision regarding the adoption of new medicines, taking into account their economic impact. This can lead to a sensible reduction of costs, increasing patients’ access to innovative drugs, and at the same time containing pharmaceutical expenditure within the national healthcare system budget [6]. This study aims at analyzing the differences between the approach of the European healthcare systems to the pharmaceutical market and the American one.

## 2. Methods

This paper highlights the opportunities and the limitations given by the application of MEAs in European countries as opposed to the American market which does not regulate pharmaceutical prices. Data were collected from the OECD, the European Medicines Agency, and the national healthcare agencies of US and European countries. A literature review was undertaken in PubMed, Scopus, MEDLINE, and Google for a period of ten years (2010–2019). The period 2020–2021 was considered in order to compare changes in pharmaceutical expenditure related to the COVID-19 pandemic. Additional material, based on data collection about MEAs, was used from European and international surveys managed by private or public organizations.

Several keywords used to define MEAs were identified through the above-mentioned initial literature review. The following keywords and terms were retained and used in the search: patient access schemes, pharmaceutical risk sharing, risk sharing schemes, risk sharing agreements, managed entry agreements, MEAs, payment by result, performance-based risk sharing agreements, coverage with evidence development, and price volume agreements. The search was limited to English-language articles.

The inclusion criteria specified that the title of the article included or referred to at least one of the searched words and concerned payment for performance and/or risk sharing agreements and/or MEA experiences across countries, including systematic or integrative reviews.

The exclusion criteria included articles about capitation (monetary allocation to doctors, physicians, nurses, and hospitals), vaccines, medical devices, diagnostic tools, hospital financial schemes, and/or pure financial schemes. Editorials, commentaries; letters to the editor; guidelines; and studies that did not identify MEAs were excluded from this study.

We initially collected 1329 articles among the selected databases. A total of 879 papers remained after removing duplicates and 163 were further selected by reading the abstract. Considering the content of the selected papers, 65 articles were read extensively but only the most representative have been mentioned in this work.

## 3. Results

### 3.1. US Healthcare System

Accounting for one-third of global sales, the US is the largest pharmaceutical market and is expected to continue to grow at an average annual rate of over 6% per year [7]. It is also the third most populous country in the world, with a population of over 338 million people. The healthcare system is one of the most complex among industrialized countries, described as a hybrid system, in which healthcare facilities are provided by the public sector (the federal government, state, and local governments), the private sector (private insurers and businesses), and the consumers (out-of-pocket expenses and self-pay). This system is the result of a combination of historical changes and maturation that too often have led to a variety of serious problems, fixed with different short-term financial performances and laws acting as a layered adjustment, which contributed to creating a complex healthcare system which, in some aspects, is inefficient and not cost-effective [8]. The inefficiency is related mainly to rising prescription drug prices and to the nation’s high healthcare costs, and even dedicated policy actions (e.g., Affordable Care Act, ACA) do not always consider the weakest segment of the population, from an economic point of view.

**Figure 3:**
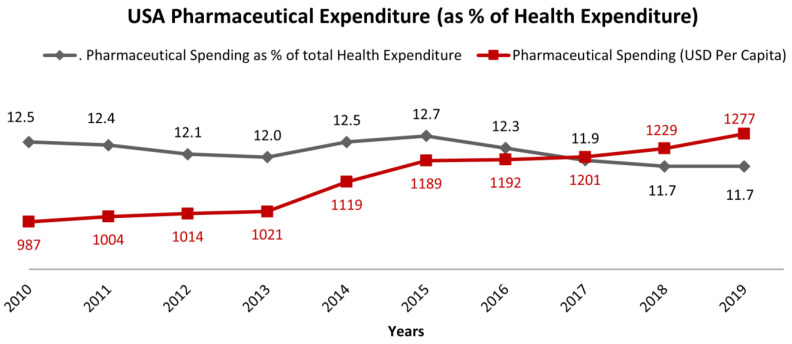
Pharmaceutical Spending as % of total Health Expenditure (pharmaceuticals in hospitals are not considered) and Pharmaceutical Spending as % of total Health Expenditure in the period of 2010–2019 [2].

Indeed, the US exhibits the highest expenditure on pharmaceuticals per capita (USD 1277 in 2019), and spent more on healthcare as a percentage of its GDP (16.7% in 2019) among OECD countries in 2019. Additionally, more than 10% of healthcare expenditure is managed by the pharmaceutical market. This disproportion, compared to other industrialized countries, largely reflects the predominant private character of the health insurance coverage in the US, with the two major public insurance programs, Medicare and Medicaid, accounting for only 40% of total health expenditure. Despite the high costs of the US healthcare system, life expectancy at birth, which was 79 years in 2021, ranks it 46th among 100 nations, with a modest improvement compared to 1990 (75.2 years, 20th among 34 nations). Additionally, there is constant growth in the prevalence of obesity, infant mortality, heart and lung diseases, and sexually transmitted infections [9]. As a matter of fact, the increasing costs of healthcare services also have important clinical implications, leading to disparities in insurance coverage, health access, efficiency, and equity. The reason for the increasing costs of healthcare can be partially ascribed to the rise in chronic diseases, high administrative expenses, and the cost of new technologies and prescription drugs. After a modest reduction of costs, mainly due to the patent expiration of several medications, in 2013 the US pharmaceutical expenditure began to increase again (Figure 3). After reaching the highest level in 2015, the expenditure started to decrease, down to 11% in 2020. At the same time, pharmaceutical spending in terms of USD per capita increased from USD 987 in 2010 to more than USD 1300 per capita in 2020. This means that the US reduced the cost of pharmaceutical expenditure within the general cost for healthcare, but this trend was not correlated to a reduction of cost for the population, moving the costs for new pharmaceutical entities directly to patients.

Some analysts have argued that “the availability of more expensive, state-of-the-art medical technologies and drugs fuels healthcare spending for development costs and because they generate demand for more intense, costly services even if they are not necessarily cost-effective” [10]. In effect, high prices of new medicines are not always justified by evident clinical benefits, giving the appearance that price is more determined by market conditions than by any real value in terms of wider advantages for patients. For the comprehension of the drug regulatory process, it is important to understand the setting of drug prices in the US, which basically consists of a restriction/absence of price negotiation by the national healthcare system.

### 3.2. US Regulatory Approval of Pharmaceutical Products

**Figure 4:**
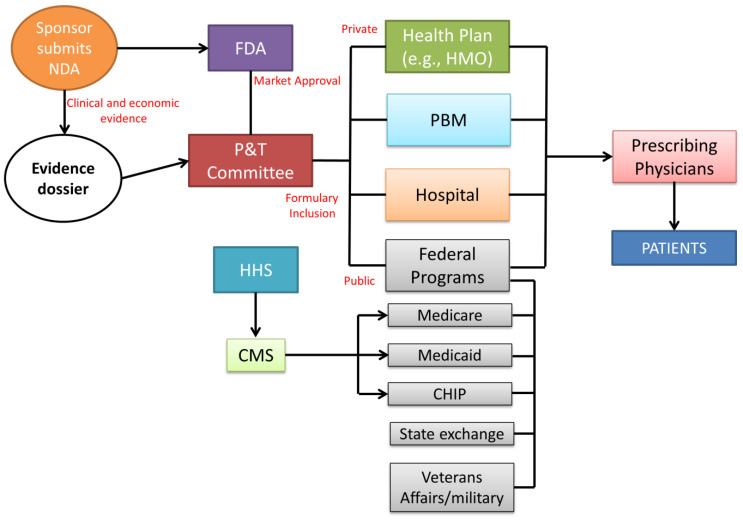
Regulatory flux for the approval of pharmaceutical products (abbreviations: NDA, new drug application; FDA, Food and Drug Administration; HHS, Department of Health and Human Services; CMS, Centers for Medicare & Medicaid Services; HMO, health maintenance organization; PBM, pharmacy benefit manager) [12].

The lack of price regulation does not imply a lack of control regarding the safety and efficacy of new therapeutic entities. Before pharmaceutical products can be marketed in the US, they are subject to market approval by the US Food and Drug Administration (FDA) [11,12] (Figure 4).

The main role of the FDA is carrying out the evaluation of pharmaceuticals and medical products by ensuring the safety and efficacy of human drugs, biological products, and medical devices before and while they are on the market. In particular, the product dossiers are evaluated by the FDA’s Center for Drug Evaluation and Research (CDER). The company must prepare an exhaustive dossier which is assessed by the CDER to determine whether the drug is ready for public utilization and if the manufacturer can apply for a new drug application (NDA) to introduce the medicine into the US market. After FDA approval, the manufacturer submits clinical and economic evidence to the pharmacy and therapeutics (P&T) committee for formulary consideration. Composed of both pharmacists and physicians, the committee examines the drug dossiers, considering drug acquisition costs and potential budget impact. The formulary inclusion influences the subsequent healthcare decision-makers (health plans, pharmacy benefit managers (PBMs), hospitals, and government agencies) to allow reimbursement and/or formulary placement of a new drug, therapy, or new formulation of an existing product [13]. Regarding the private sector, coverage decisions are customized per individual health plan. Private health insurers use evidence dossiers and collect their own clinical and economic data to determine coverage for a new therapy. Hospitals also create evidence dossiers through their own P&T committee to develop and manage the hospital formulary as a tool for prescribing treatment to patients. In drug prescription coverage, the public sector is largely represented by the Centers for Medicare & Medicaid Services (CMS). Based on the P&T committee formulary decisions, coverage of drugs used during inpatient or outpatient procedures or hospitalization for the public federal coverage is included within the reimbursement provided for a specific therapy. Health plans maintain a formulary of drugs suitable for coverage within a particular insurance policy (co-payment or co-insurance system). For drugs not included in the formulary, a patient has to pay 100% of the drug cost. The decision-making process does not require a federal HTA. However, Medicare periodically requests that the Agency for Health Care Quality (AHRQ) review new treatments or procedures for efficacy, safety, and comparative effectiveness. Payers in the US, the public Center for Medicare & Medicaid Services (CMS), and private insurance companies do not regulate the price of a pharmaceutical product, allowing the manufacturers to set prices freely. The lack of a federal body for pricing negotiation involves a variety of factors that influence the final price, building a complex system that can weigh down healthcare professionals and mostly the patients [14]. In fact, the price estimation in the US is the result of a supply chain that involves mainly three transaction areas: from manufacturer to wholesaler, from wholesaler to pharmacy, and from pharmacy to patient [15]. Several policies at different levels of government, joined with a lack of national control, induced the creation of a large number of drug prices, which have further complicated the pharmaceutical market and drug accessibility. Among them, the Federal Upper Limit (FUL) is a pricing guideline used by CMS to determine the maximum reimbursement amount for certain generic drugs. The FUL is based on the average wholesale price (AWP) of a drug, which is a benchmark price used in the pharmaceutical industry to determine the cost of a prescription drug. The AWP is the estimated average price that a wholesaler would charge a retailer for a drug before any discounts or rebates. Another drug price is the Wholesale Acquisition Cost (WAC), related to the price at which a pharmaceutical company sells a drug to wholesalers or distributors. It is the starting point for determining the price of a drug in the supply chain and is used as a benchmark for determining the cost of drugs for healthcare providers and insurance companies. The Average Manufacturer Price (AMP) is a measure of the average price that a pharmaceutical manufacturer receives for a prescription drug, including any discounts or rebates offered to wholesalers, pharmacies, and other buyers. The list of prices continues with the Average Sales Price (ASP), which is a measure of the average price that a pharmaceutical company receives for a prescription drug, including any discounts or rebates offered to wholesalers, pharmacies, and other buyers. The Estimated Acquisition Cost (EAC) in drug pricing refers to the projected cost of purchasing a drug or a medical device, considering all factors such as bulk discounts, rebates, and other discounts that may be applied, whereas the Average Actual Cost (AAC) is a measure of the actual cost of a drug or medical device after it has been purchased and used. The AAC is calculated by dividing the total cost of the drug or device by the number of units purchased, providing a more accurate representation of the true cost of the drug. Finally, the amount reimbursed to the pharmacy to cover the charge for professional services and overhead costs is called Dispensing fee [16].

**Figure 5:**
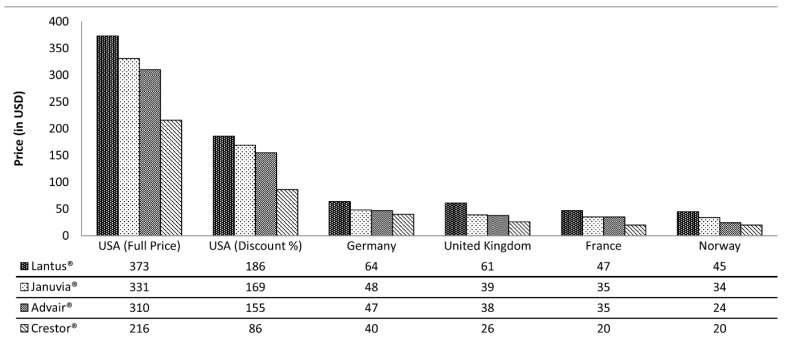
Comparison between four different drug prices, before and after an estimated discount applied in the US market, compared with the price set in four European countries [19].

However, payers are allowed to set the reimbursement price/rate. This agreement differs between the public and the private sector, establishing if the drug price has to be covered entirely by the payers or if the patients have to cover some part of the prescription cost, in a sort of co-payment modality. To date, private payers have exploited MEAs, but considering the confidentiality of negotiations, it is not possible to assess the evaluation of quality and efficiency. Medicare is the CMS sector which in recent years adopted a different kind of agreement in the United States. These initiatives largely focus on devices and surgical procedures rather than drugs. Only a few of these negotiations are focused on medicines [17]. Coverage with evidence development is used as there are no explicit agreements between the manufacturer and the payer, but there are implicit assumptions that the data will be used for future coverage decisions [18]. However, even though the reimbursement process is applied in the US market, the price of pharmaceuticals is more expensive compared to other industrialized countries. Among all the four drugs analyzed in Figure 5, the price is much higher in the US than in the European countries considered, even after the estimated discounts [19].

### 3.3. The Affordable Care Act (ACA)

The ACA, also known as Obamacare, is recognized as the most colossal change in the USA healthcare system since the reform signed by Lyndon B. Johnson in 1965, consisting of the creation of Medicare and Medicaid, the two major public health services in the USA [20]. The ACA was signed by President Obama in 2010 and it aims to increase insurance coverage, improve quality of life, control healthcare costs, and provide access to healthcare services for US citizens [21].

The ACA caused an increasing number of insured individuals while the achievement of a reduction in healthcare costs as well as improvement in care quality is questionable.

In the US healthcare system, the ACA has been highly criticized because of quality, coverage, and costs, being classified as the worst in terms of impartiality, effectiveness, prices, and tangible results. Moreover, the high frequency of “un-insurance”, with patients directed to walls to care, and financial uncertainty, have increased the incidence of preventable deaths [22]. More specifically, the ACA is based on:

- a mandate for people to have an appropriate level of health insurance;
- federal subsidies for about 34 million people to contribute to health insurance payments, subsidized through Medicaid and exchanges;
- regulations on the exercise of medicine.

The major impact of the ACA is related to the growing authority regulation of the Independent Payment Advisory Board (IPAB) and the Patient-Centered Outcomes Research Institute (PCORI), associated with discounts on medical doctor reimbursement. However, most believe that the ACA does not supplement the funds of the USA’s public or private healthcare system, suggesting an increasing deficit over a reduction [20].

### 3.4. European Pharmaceutical Market

The European pharmaceutical market is a complex scheme regulated by different national healthcare systems, under the common managership of the European Medicines Agency (EMA). National healthcare organizations across Europe attend to more than 500 million people in health services, trying to guarantee equal access to the population, increasing the availability of healthcare products and the general quality of life. Health expenditure in Europe accounts for a significant portion of GDP, ranging from 5.5% in Latvia to 11.4% in Switzerland in 2015, posing a serious challenge for payers in budget management (Figure 1). Taking into account the final year of the examined period, 2019, Latvia maintained the same percentage of GDP, whereas the countries with the highest health expenditure were France and Germany, with 11.1% and 11.7% of the GDP, respectively [23]. The influence of COVID-19 in the increase in health expenditure is described in a separate section.

In terms of pharmaceutical spending per capita, in the same year Denmark and Estonia handed out USD 342, while the European country with the highest expenditure was Switzerland with USD 1 056 (Figure 1). In 2019, Denmark was confirmed to be the lowest ranked country in terms of pharmaceutical spending per capita, but in the opposite direction Switzerland reduced pharmaceutical spending per capita to USD 826, and Germany had the highest expenses, ranging from USD 756 to USD 880.

Pharmaceutical spending is one of the most important expenditure items among health costs, accounting for between 7% (Denmark, the Netherlands, Norway) and 28% (Hungary and Greece) of the total health expenditure in 2019 (Figure 2). In recent decades, national healthcare services have applied several cost-containment policies to keep health expenditure under control [24,25]. These proceedings have been practiced even more since the global economic crisis in 2008. Indeed, healthcare systems across Europe pushed for containment policies in order to reduce health costs, implementing or planning more than 116 general health reforms between 2010 and 2011 alone [26]. Nevertheless, European payers believe that the main budget driver in health services is drug expenditure. For this reason, national healthcare organizations exploited new or improved tools able to analyze the real efficacy of new therapies in relation to the budget impact, optimizing the distribution of their scarce resources in the most efficient way [27]. Each country is free to set its own national pricing and reimbursement policy as well as to implement regional regulations, allowing the creation of a wide variety of pharmaceutical agreements. These are based on the social environment, historical period, education, gender, population aging, physical environment (water, air, pollution), and population behavior (sport, smoking, alcohol, food education). In recent years, among different cost-containment policies (price cuts, alteration of co-payment ratio, more rational prescription), European countries have applied external reference pricing (ERP) as a widespread tool for the reduction of pharmaceutical costs. In 2013, out of 31 European countries (28 EU Member States, Iceland, Norway, and Switzerland), 29 countries (except Sweden and the UK) use ERP to set the price of new drugs and/or as supplementary decisional criteria in the pricing and reimbursement process [28]. For a long time, this method was one of the main cost-containment tools adopted by national healthcare services, in which the price of a medicine in one or several countries became the benchmark or the reference price in setting or negotiating the cost of a pharmaceutical product in another country.

On the other hand, national healthcare organizations may operate differently among countries, retrieving information about the value of a pharmaceutical product not only based on clinical evidence, safety, and efficacy but also related to the budget impact, epidemiologic data, and demand-side measures. Policy-makers, after the approval from medicine regulators, will negotiate the drug price based on clinical evidence of that country, which does not always fit with the needs of other states. Then, if the price for a given product changes in one country, this may affect the price in others, with potential disparities among populations. In recent years, the strategy of sharing prices among nations has been successfully flanked by the application of MEAs [29]. Using MEAs, payers and manufacturers are involved in the sharing of the risk for the development and commercialization of new drugs. HTA bodies take a fundamental role in the risk sharing analysis. Indeed, the majority of nations have established evidence and justifications about the costs of drugs by the use of HTAs as an instrument to inform and lead decisional bodies during and after the price definition [30]. An HTA is a multidisciplinary process able to describe, analyze, and judge health technologies based on clinical, ethical, and economic evaluation in the short and long term. The aim is to achieve the best value for new health technologies, compared with previous treatments, and in this way lead health decisional bodies towards a better therapy evaluation, considering patient needs related to the pharmaceutical expenditure. In this way, insufficient evidence at market launch, linked to the need to limit the budget impact of new health therapies, is under the strict control of clinical evidence provided by HTA and cost-effectiveness analysis, to keep the expected benefit of innovative drugs as realistic as possible after pharmaceuticals are placed on the market. Payers agree to cover promising health technologies on a conditional basis, while evidence of real-world effectiveness and cost-effectiveness is evaluated from a budget-restraint perspective. Health decisional bodies have a crucial role to identify powerful and innovative products, compared to previous treatments, to improve patient outcomes. From the pharmaceutical manufacturer’s point of view, the creation of a powerful and convincing dossier for an innovative product is a fundamental step toward market access. A lot of efforts must be put into planning a positive frame that supports the uptake of a new product, communicating the value not only from the point of view of efficacy and safety but also regarding non-clinical positive attributes, such as the impact on quality of life, potential cost saving, and ease of administration [31]. Producers may also consider the impact and the implications the product may have on the wider healthcare market.

Nowadays, the old paradigm about the innovativeness of new drugs is not enough, they must show value above and beyond available treatments.

### 3.5. Managed Entry Agreements

The increasing availability of potentially life-saving high-cost drugs and increasing patient expectations have pressed payers and medicine regulators to include new therapies in reimbursement lists, even when data and the overall evidence base available at registration are often insufficient to estimate additional benefits related to current therapies and to the budget impact in real life. Clinical trials before access to the market leave residual but important uncertainties from the payers’ point of view [32]. Different kinds of uncertainties—uncertainty around clinical evidence, cost effectiveness and budget impact, uncertainty around the price, and eligible patient population—may delay reimbursement decisions and patient access. It is important to underline the differences between uncertainty and risk. Toumi affirmed that “Uncertainty is a situation in which the current state of knowledge is such that the order or nature of things is unknown: consequences, extent or magnitude of circumstances, conditions, or events are unpredictable and credible probabilities to possible outcomes cannot be assigned. In contrast, the risk is characterized by at least possible outcome(s) that are identified and a probability for the possible outcome(s) can be assigned” [33]. Assessing the risk allows payers and manufacturers to manage price regulation and reimbursement of new pharmaceuticals in a wide range of risk sharing schemes. MEAs are defined “as arrangements between a manufacturer and payer/provider that enables access to (coverage/reimbursement of) a health technology subject to specified conditions. These arrangements can use a variety of mechanisms to address uncertainty about the performance of technologies or to manage the adoption of technologies to maximize effective their use or limit their budget impact” [34]. More specifically, MEAs or conditional reimbursement agreements are contracts between pharmaceutical companies and regulatory agencies that are useful to regulate introduction to the market of novel drugs on a conditional basis. These agreements represent a method of assigning the price of new drugs, that can be considered dynamic, since it is dependent on the benefits demonstrated in randomized controlled trials (RCTs) and they need to be monitored in real life. This could be of crucial importance in long-term diseases such as Parkinson’s or Alzheimer’s, where the advancement of new therapies needs long trials to assess their potential efficacy [35]. These agreements can also be based on the consumption volumes. Therefore, for the former, the cost of the drugs can be related to their real effectiveness, based on a priori established rules, while, for the latter, it becomes inversely proportional to the number of treated subjects.

In more detail, in the case of price–benefit agreements, there are payment by result (PbR), risk sharing (RS), and success fee (SF) agreements. In the case of the costs according to consumption, cost sharing (CS) and capping agreements can be considered [36]. Each agreement depends on the drugs, pathologies, and pending requests. Starting from 2006, Italy was the first country to assume this methodology and since this date the number of active compounds has significantly increased.

An example of a price–volume agreement is drugs used for hepatitis, that were introduced in the market with high prices, but received conspicuous discounts based on the treated population.

To make the agreements applicable, it is necessary that there is a single monitoring system which is ensured by AIFA, which clinicians and pharmacists can access, respectively, during the prescription and dispensing phase. This system, in addition to providing economic sustainability for early access to novel, innovative treatments, could become a useful tool for verifying drug effectiveness in real life. The monitoring system used by the AIFA registers, underlying the implementation of the MEAs, is employed for the analysis of prescriptions useful for improving clinical practice [37].

**Figure 6:**
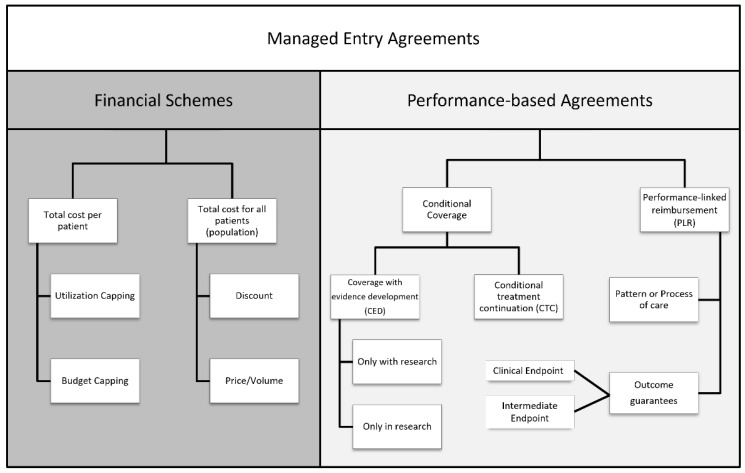
The taxonomy of Managed Entry Agreements (MEAs) is divided into two main forms: Financial schemes and performance-based agreements.

In recent years, the application of MEAs has increased across Europe. Compared to 2009, between 2010 and 2013 the number of schemes nearly doubled [38]. From 2013 to 2016, the number of agreements suffered a strong reduction, with a maximum of 437 agreements in 2016, although only 42% of these were categorized as active [39]. Different and distinct forms of agreements have been implemented across different countries, with two main types: financial schemes and performance-based schemes (Figure 6) [40].

**Figure 7:**
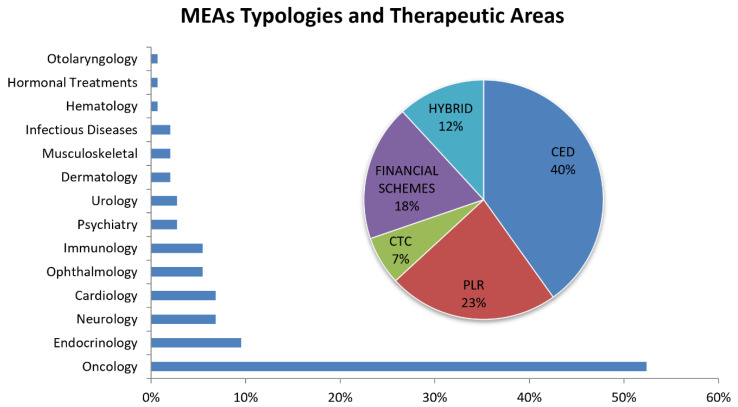
Bars chart: Percentage of arrangements developed in different therapeutic areas. Pie chart: Percentage of performance-based arrangements in 2013. CED: coverage with evidence development; CTC: conditional treatment continuation; PLR: performance-linked reimbursement.

Financial schemes try to manage the budget impact with the application of various non-outcome-based agreements, differentiated by if they consider the total cost per patient or the total cost for all patients (population). These tools are based on the value of a new therapy and its financial impact without the examination of clinical evidence, but with the focus on controlling financial expenditure with pharmaceutical companies refunding in over-budget situations (price volume agreements, PVAs), or involving the use of free drugs once patients have exceeded an agreed utilization limit to enhance reimbursement/funding with finite resources (price capping). On the other side, performance-based schemes are founded on data efficacy, effectiveness, and safety, allowing payers and manufacturers to set penalties or rewards (higher or lower price, reimbursement) after the analysis of the clinical performance of the product in the real world [41]. Specifically, performance agreements can be divided into two categories: (i) conditional coverage, in which coverage is granted on the initiation of a program of data collection that informs the use of the medical product in the payer population; and (ii) performance-linked agreements, where the reimbursement level for covered products is linked to the measure of clinical outcomes. Conditional coverage can be further divided into two categories. The first one is the coverage with evidence development (CED), in which a binary coverage decision is influenced by the collection of additional population-level evidence to support the continuation, extension, or even withdrawal of the agreement. Coverage may be also conditioned on individual participation in research or a scheme to perform studies for that specific therapy in the payer patient population. The second category under conditional coverage is the conditional treatment continuation (CTC), where the continuation of coverage for individual patients is conditioned upon meeting short-term treatment goals. The performance-linked reimbursement category is also divided into two parts: (i) the guarantee of the outcome, in which the pharmaceutical company provides rebates, refunds, or price adjustments if their product fails to meet the agreed-upon outcome targets; and (ii) the pattern or process of care, in which reimbursement is linked to the impact on clinical decision making or practice patterns. European countries applied several types of MEAs in different medical fields. Carlson et al. [38] identified more than 140 agreements until 2013, among which more than 88% were settled between 2003 and 2013, the period in which MEAs accounted for a good percentage of pharmaceutical price agreements. Figure 7 shows the percentage of MEAs based on the therapeutic area (bars) and type described in the previous taxonomy (pie chart).

More than 50% of agreements have been applied to anticancer therapies, followed by endocrinology at 9.5% and neurology at 6.8%. The major application of cost-containment policies such as MEAs is in the oncological area, where single treatment cycles can be very costly, and at the same time, clinical data at market approval are usually inadequate or not exhaustive enough to thoroughly describe their effectiveness and cost effectiveness.

Regarding the MEA types, more than one-third of agreements are CED (40%), followed by PLR (23%), financial schemes (18%), hybrid (12%, in particular: PLR–CTC, CED–PLR, PLR financial schemes, PLR–CTC–financial schemes, CED–PLR–financial schemes) and CTC (7%). CED and PLR are the most employed schemes (more than 60% of MEAs examined) due to the coverage of pharmaceutical products for which additional data—gathered in the context of clinical care and with a certain degree of uncertainty—would further clarify in terms of impact on the health of patients in the real world. In particular, PLR schemes help in the management of new entry therapies by checking short-term treatment goals for individual patients (e.g., list of clinical endpoints). In case of negative outcomes, the manufacturers must cover the cost by reimbursing the payer, or by covering the same amount of costs for another patient. In this way, pharmaceutical products that did not completely convince payers and healthcare bodies can have a chance to be commercialized, and despite the high price, they can also be accessible to patients due to the reimbursement agreements set by national healthcare services. The commercialization phases for pharmaceutical products in the US and in Europe are not easy to compare, due to different processes and different decisional steps that European countries adopt before a new therapy is authorized to be on the market, with the relative pricing and reimbursement phase. Ferrario and Kanavos [42] presented an important report on behalf of the European Medicines Information Network (EMI-net) about MEAs and the European experience, reaffirming the positive trend European national healthcare services show in the application of pharmaceutical agreements. Based on the previous survey, Italy and the respective national healthcare services presented a higher rate of MEAs, with a positive experience in managing drug prices related to the cost-containment policies. The price and reimbursement regulation of pharmaceutical products adopted in Italy could be representative of the MEA application process in Europe.

### 3.6. The Italian Experience

The major healthcare payer in Italy is the National Health Service (Servizio Sanitario Nazionale, SSN), which provides universal coverage for citizens and residents. The SSN reimburses pharmaceuticals listed on a national drug formulary managed by the Italian Medicines Agency (Agenzia Italiana del Farmaco, AIFA). AIFA assesses the value of interventions based on scientific, clinical, and economic data and negotiates with pharmaceutical manufacturers to establish drug prices and risk sharing agreements. The negotiation activity that AIFA carries out on this basis is regulated by Italian law (Law n. 326 of 24 November 2003), and the methods and criteria are set out in the Interministerial Committee for Economic Planning (Comitato Interministeriale per la Programmazione Economia, CIPE) Resolution of 1 February 2001 [43].

**Figure 8:**
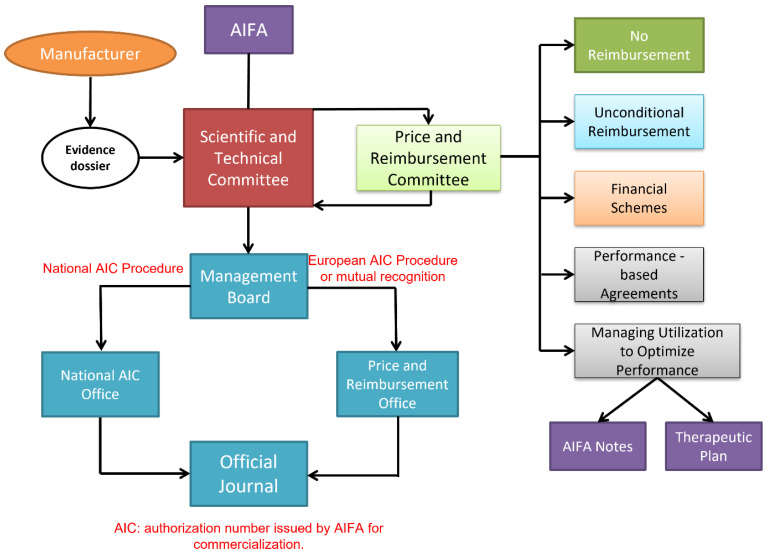
The Italian price and reimbursement process and the panel of different managed agreements that are available.

As shown in Figure 8, the first step is the submission of an evidence dossier by the pharmaceutical company to the AIFA Scientific and Technical Committee, demonstrating a positive cost/benefit ratio and advantages useful for the treatment of pathologies for which there is no effective cure, or greater effectiveness than medications already available for the same therapeutic indications.

The new therapy must also be of interest to the SSN, such as by having significant clinical superiority over existing products or at least equal effectiveness and safety compared to other products already available, with a sensible cost reduction. At this point, the AIFA Price and Reimbursement Committee examines the price and reimbursement claims received, also supported by the consumer information and expenditure data provided by the Medicines Utilization Monitoring Centre (OsMed). During the meeting, contractors reach an agreement on the various applications that have been recorded by an Italian marketing authorization (national or European marketing authorization procedure) by fixing the prices and the conditions of reimbursement. The Price and Reimbursement Committee sends back the result of the negotiation to the Scientific and Technical Committee, which analyzes the price and judgment expressed and submits the dossier to the examination of the AIFA Management Board for the subsequent resolution, which is then published in the official journal of the Italian Republic [44].

The Italian drug reimbursement landscape is characterized by a wide range of options: no reimbursement, unconditional reimbursement, or reimbursement in the frame of the MEA instruments analyzed before. AIFA notes and therapeutic plans are examples of country-specific MEAs used to manage budget impact and uncertainty around clinical and cost effectiveness. Reimbursements are limited to specific patient sub-groups. The AIFA note is reported by the general practitioner on the prescription form, and this allows the patient to obtain the medicinal product free of charge.

Moreover, the Italian healthcare system matches the use of performance-based agreements with data collection as part of the monitoring registries. These registries track patient eligibility and subsequent enrollment, drug prescription, patient assessment and/or outcomes, and reimbursement requests for non-responders. Collecting data on treatment performance is critical to understand how the drug performs in real life and is used in clinical practice [45]. In Italy between 2006 and 2009, there were 6 oncology MEAs, and between 2010 and 2013 they tripled, reaching 18 schemes, highlighting the importance of these agreements to make expensive new generation anticancer drugs more patient accessible [46]. The Italian situation is atypical if compared with other European countries, since the number of agreements increased remarkably, from the above-mentioned 18 schemes of 2013 to 85 agreements reached in 2016 [39].

### 3.7. The COVID-19 Pandemic

Due to the high degree of confidentiality of information related to the COVID-19 pandemic and the drugs adopted between 2020 and 2021, for vaccines, we cannot indicate any MEAs that the different nations could have adopted with manufacturers, but we can indicate how parameters such as health expenditure related to GDP and general pharmaceutical expenditure data have varied before and after the viral epidemic. A more recent example is represented by the COVID-19 therapies based on remdesivir, molnupiravir, and PF-07321332/ritonavir as their use is subject to prescription and administration cards with simultaneously sent data of the carried out therapies.

Regarding health and pharmaceutical spending during the COVID-19 pandemic, it is useful to describe the situation in the previous period, 2010–2019 [23]. For OECD countries, the average health expenditure as % of GDP was 8.7% in 2019, whereas for the US the same parameter was at 16.7%. In 2010, the first year of the analyzed period, the health expenditure of the US was 16.2% of the GDP; this means that the increase in the expenditure in ten years was 0.5% of the whole GDP. As regards European countries, in 2010 the average health expenditure was 8.4% of the GDP in 2010, with an increase of 0.3% in ten years. Due to the emergency caused by the uncontrolled SARS-CoV-2 epidemic, in 2020 the health expenditure increased by 1% in the OECD area and 2.1% for the US, reaching 9.7% and 18.8% of the GDP, respectively [2].

The OECD database for the year 2021 is not complete for the whole OECD area, with some representative countries which communicated the information such as Italy or Germany [23]. In the case of these two nations, the health expenditure followed the same trend in the period 2020–2021. A conspicuous increment in the health expenditure was observed between 2019 and 2020, 1% of GDP for Italy and 1.1% of GDP for Germany, followed by a slight contraction of the expenditure in 2021 for Italy (9.45% of GDP, −0.15%), and a constant value for Germany. This trend is probably due to the still present need to invest a good part of public money in health due to the COVID-19 pandemic, resulting in a plateau or even an increase in GDP spending for several countries as long as the pandemic situation continues.

Regarding the pharmaceutical expenditure per capita, in 2019 the US spent USD 1277 per capita, with an increase of USD 290 compared to 2010, whereas for the other OECD countries the spread between 2010 and 2019 was less than USD 70 (from USD 505 to USD 571).

During the COVID-19 pandemic period, in the US the pharmaceutical expenditure increased to USD 1310 per capita, whereas for European countries the expenditure was constant (data in 2020, those for the year 2021 are incomplete) [2].

This information gives us an idea of how, in European nations, individual governments have taken on the expenses of the emergency due to COVID-19, having little or no impact on the spending of the population, unlike the US where a certain increase in spending has been observed. The strengthening of the national health service, from a structural point of view with the increase in the number of doctors on duty and the strengthening of the COVID-19 wards, together with the distribution of drugs and vaccines for the containment of the pandemic, has led to an increase in health expenditure in terms of GDP for both European countries and the US. Meanwhile, regarding the cost of individual patients, only the US saw an increase, while European countries were able to avoid directly burdening the population with the costs. This increase is possibly attributable to a private US healthcare system with a free drug price not subjected to a negotiation.

## 4. Discussion

European countries apply different decisional processes over time to adopt MEAs. Compared with the Italian process, all these countries present the common instruments described before, such as dedicated sections of their national health systems, the use of HTA bodies, and a specific decisional flux which contributes to the price and reimbursement negotiation for the obtainment of pharmaceutical agreements. The US does not have a central agency that negotiates prices and reimbursement with hospitals and drug manufacturers. The P&T committee develops and manages the formulary system using clinical and economic dossiers as reference documents for decision making, along with other key considerations, but without federal HTA requirements. Insurance agencies negotiate individually with hospitals, doctors, and manufacturers to set their own prices; insurers do not get a bulk discount. A fragmented system means that Americans pay more for every type of therapy (Figure 5). On the other hand, the lack of a centralized regulatory process makes new medicines available in the US within less than two months and halves the time to approval, whereas drugs available for the EU market are subject to significant delays, of almost one year, due to national and regional regulatory processes, HTA evaluation, and budget-restraint challenges [47]. In terms of pharmaceutical access, the US healthcare system can be defined as the most advanced in the world, the leader in the application of new and innovative therapies, and more efficient compared with European health systems. Indeed, due to bureaucratic delays, difficulties in negotiation, or, in the worst case, failure of negotiation, European countries prolong the use of old therapies, with a high rate of hospitalizations and deaths before a new drug is available. However, the entry delay is rewarded by a wider coverage of patients who can obtain access to a new therapy for free, independently of social class or economic wellbeing. Disparities in access to new and costly therapies are some of the most important challenges for the US health system. In 2014, nearly 33 million people in the US had no health insurance [48]. The Center for American Progress estimated in 2009 that the lack of health insurance in the US costs society between USD 124 billion and 248 billion per year [3]. Moreover, the continuous rise in private health insurance premiums (more than 50% for family coverage from 2005 to 2015), the constant growth of co-payment solutions for new therapies, and the payment of co-insurance for certain specialty drugs lead to clinical and economic consequences for American families. If the US health system makes no major structural changes, the average cost of a family health insurance premium will surpass the average household income by the year 2033 [49].

A 2014 survey of bankruptcies found that between 2005 and 2013 medical bills were the single largest cause of consumer bankruptcy, with between 18% and 25% of cases directly caused by medical debt [50]. The prohibitively high cost of drugs also leads patients to avoid prescriptions because of the cost, prolonging the risk of pandemics, reducing productivity, and driving up healthcare costs. Non-adherence due to all causes has been estimated to contribute to USD 105 billion in avoidable healthcare costs annually [51]. The Medicare prescription drug benefit, also called Medicare Part D, prompts the application of discounts and agreements, but only 15.8% of the whole US population receives this coverage from the government. Moreover, the Medicare system is partially paralyzed by the poor quality of scientific studies that did not meet its needs for coverage determination [18]. In particular, the elderly and disabled, who constitute the patients covered by the Medicare program, were often under-represented in clinical trials. In addition, most trials failed to compare treatments with currently covered alternatives, and the outcomes reported in trials were typically short term, being of limited importance to patients or their clinicians. CMS needs to generate more relevant evidence for coverage decisions, applying its payment authority to promote the development of evidence designed for specific aims [52]. In general, the US healthcare system is affected by historical and structural problems, due to the sustainability of such a big system (more than 300 million people), the private interests, laws, and local measures which create a barrier to the implementation of a national single-payer healthcare system. Additionally, if an HTA body is introduced in the US, it could positively influence the management of health resources on the basis of improved cost-effectiveness analyses. The subsequent regulation of pricing and reimbursement decisions would lead to greater control of the pharmaceutical companies, with the consequent loss of the free market pricing. Conversely, some experts support the American health system, arguing it is possible to deliver many new medications to patients while still managing costs because the system relies on competitive markets to set prices and encourage innovation. The introduction of new medications into the market is progressively characterized by competition from other brand-name and generic drugs in the same therapeutic class, driving down price and thus increasing the patient accessibility. Moreover, they affirm that in European countries the control of the market access using national cost-effectiveness policies has created barriers to patients’ access to many important treatments [46]. Although this aspect of drug accessibility is correct, the presence of innovative high-price therapies that can be used only by a small percentage of patients brings severe affordable care challenges for the US population. MEAs as instruments for health cost containment have increased drug accessibility in European countries, giving the whole population the possibility to receive equal treatment. It is important to underline that MEAs are not a perfect tool. Confidentiality of negotiation which prevents a complete overview of the cost-containment measures, the possibility of negotiation failure which can reduce the spread of treatments available for a given disease, the direct control of the drug market by the national health services, and the different approaches that each country can apply for the same medication can discourage the adoption of MEAs. The complexity of MEAs and the misguided or miscommunicated goals and strategies mean that MEAs are often not properly executed. Nevertheless, these drawbacks do not negate the positive impact that this kind of agreement can have on the national healthcare budget and the population’s access to new therapies [40].

Limitations on this work are related to the inability to find all relevant sources related to MEAs since different names may be used before the adoption of a standardized taxonomy. The papers considered were not always related to the exact year of publication, and a large part of the information related to pricing and the reimbursement process is not transparent in many countries, and therefore it is difficult to obtain detailed reports and procedures. Finally, the number of peer-reviewed articles being published each year may not completely reflect the level of interest in MEAs. However, such a comparison and in-depth description of the decision-making processes related to the introduction of new drugs to national markets provide a considerable contribution to our knowledge of the sector.

## 5. Conclusions

Most European countries are making use of MEAs to reduce the lack of information about cost effectiveness, access, and budget impact. Cost-containment policies, due to continuous health budget cuts, mostly after the economic crisis, have reduced the ability of nations to increase the resources for drug access. In the US, both private and public payers can freely decide to draw up an MEA with a pharmaceutical industry or not. The increasing interest in reducing health costs without compromising safety and quality is leading the US market to innovative approaches to commercialization and pricing. More and more insurance agencies are making use of MEAs, with increasing attention on value-based agreements. Delays in the European market access suggest a direct relationship with an increasing level of scrutiny by HTA agencies and other price-setting bodies, such as in Italy. The US system allows more rapid access to innovative new therapies, with a clear benefit for patients’ health. At the same time, patient access is strongly regulated by budget availability or the high premium that families must pay for private insurance. MEAs have proved to be powerful instruments for both pharmaceutical industries and national healthcare systems, lowering market access to potentially innovative therapies with negative cost effectiveness. On the other hand, MEAs’ diversity and complexity, due to expenditure management policy, suggest that improvements should be made to the common rules of the value of effectiveness and budget impact, pricing and reimbursement, and accessibility, as well as for expenditure management policy. Furthermore, economic aspects influence most of the decisions about the process of market access to the detriment of efficacy and clinical effectiveness. MEAs should have a pivotal role in the process of market access to innovative therapies which begins with horizon scanning activities, HTA assessment, pricing, and reimbursement, and goes on with post-marketing studies.

## Data Availability

Not applicable.

## References

[1] OECD (2015). Focus-Health-Spending-2015. OECD Health Statistics.

[2] OECD (2022). Pharmaceutical Spending (Indicator).

[3] The Department for Professional Employees, AFL-CIO (2016). The U.S. Health Care System: An International Perspective. Fact Sheet 2016.

[4] Wilson J., Shah S. (2015). Pharma Market Access Success: Shifting the Dialogue from Price to Value through Strategic Communications.

[5] Carone G., Schwierz C., Xavier A. (2012). Cost-Containment Policies in Public Pharmaceutical Spending in the EU.

[6] Vogler S., Paris V., Ferrario A., Wirtz V.J., de Joncheere K., Schneider P., Pedersen H.B., Dedet G., Babar Z.U.D. (2017). How Can Pricing and Reimbursement Policies Improve Affordable Access to Medicines? Lessons Learned from European Countries. Appl. Health Econ. Health Policy.

[7] Keehan S.P., Stone D.A., Poisal J.A., Cuckler G.A., Sisko A.M., Smith S.D., Madison A.J., Wolfe C.J., Lizonitz J.M. (2017). National health expenditure projections, 2016–2025: Price increases, aging push sector to 20 percent of economy. Health Aff..

[8] Murray C.J.L., Frenk J. (2000). A framework for assessing the performance of health systems. Bull. World Health Organ..

[9] Murray C.J.L. (2013). The State of US Health, 1990-2010. JAMA.

[10] Beamesderfer A., Ranji U. (2012). “U.S. Health Care Costs.” Background Brief.

[11] AMCP Task Force on Drug Payment Methodologies (2007). AMCP Guide to Pharmaceutical Payment Methods. J. Manag. Care Pharm..

[12] Khachatryan A., Patel D. United States—Pharmaceutical. https://www.ispor.org/HTARoadMaps/USPh.asp.

[13] Walkom E., Robertson J., Newby D., Pillay T. (2006). The role of pharmacoeconomics in formulary decision-making. Formulary.

[14] Kesselheim A.S., Avorn J., Sarpatwari A. (2016). The High Cost of Prescription Drugs in the United States. JAMA.

[15] Danzon P.M. (2014). Pricing and Reimbursement of Biopharmaceuticals and Medical Devices in the USA.

[16] Mattingly J. (2012). Understanding Drug Pricing. US Pharm.

[17] CMS (2006). National Coverage Determinations with Data Collection as a Condition of Coverage: Coverage with Evidence Development.

[18] Mohr P.E., Tunis S.R. (2010). Access with Evidence Development. Pharmacoeconomics.

[19] Langreth R., Migliozzi B., Gokhale K. The U.S. Pays a Lot More for Top Drugs Than Other Countries. http://www.bloomberg.com/graphics/2015-drug-prices/.

[20] Carrasco-Aguilar A., Galán J.J., Carrasco R.A. (2022). Obamacare: A bibliometric perspective. Front. Public Health.

[21] Finegold K., Conmy A., Chu R.C., Bosworth A., Sommers B.D. (2010). Trends in the U.S. Uninsured Population, 2010–2020.

[22] Manchikanti L., Helm Ii S., Benyamin R.M., Hirsch J.A. (2017). A Critical Analysis of Obamacare: Affordable Care or Insurance for Many and Coverage for Few?. Pain Physician.

[23] OECD (2022). Health Spending (Indicator).

[24] Cook J.P., Vernon J.A., Manning R. (2008). Pharmaceutical risk-sharing agreements. Pharmacoeconomics.

[25] Ess S.M., Schneeweiss S., Szucs T.D. (2003). European healthcare policies for controlling drug expenditure. Pharmacoeconomics.

[26] Koch M.A. (2015). Pharmaceutical Market Access: Current state of affairs and key challenges—Results of the Market Access Launch Excellence Inventory (MALEI). J. Mark. Access Health Policy.

[27] Adamski J., Godman B., Ofierska-Sujkowska G., Osińska B., Herholz H., Wendykowska K., Laius O., Jan S., Sermet C., Zara C. (2010). Risk sharing arrangements for pharmaceuticals: Potential considerations and recommendations for European payers. BMC Health Serv. Res..

[28] Rémuzat C., Urbinati D., Mziughi O., El Hammi E., Belgaied W., Toumi M. (2015). Overview of External Reference Pricing systems in Europe. J. Mark. Access Health Policy.

[29] Jarosławski S., Toumi M. (2011). Market access agreements for pharmaceuticals in Europe: Diversity of approaches and underlying concepts. BMC Health Serv. Res..

[30] Ciani O., Jommi C. (2014). The role of health technology assessment bodies in shaping drug development. Drug Des. Devel. Ther..

[31] Walker S., Sculpher M., Claxton K., Palmer S. (2012). Coverage with evidence development, only in research, risk sharing, or patient access scheme? a framework for coverage decisions. Value Health.

[32] De Pouvourville G. (2006). Risk-sharing agreements for innovative drugs: A new solution to old problems?. Eur. J. Health Econ..

[33] Toumi M. (2017). Gap between Payers and Regulators. Introduction to Market Access for Pharmaceuticals.

[34] Klemp M., Frønsdal K.B., Facey K. (2011). What principles should govern the use of managed entry agreements?. Int. J. Technol. Assess. Health Care.

[35] Cacciatore I., Ciulla M., Marinelli L., Eusepi P., Di Stefano A. (2018). Advances in prodrug design for Parkinson’s disease. Expert Opin. Drug Discov..

[36] Garrison L.P., Towse A., Briggs A., de Pouvourville G., Grueger J., Mohr P.E., Severens J.L., Siviero P., Sleeper M. (2013). Performance-Based Risk-Sharing Arrangements—Good Practices for Design, Implementation, and Evaluation: Report of the ISPOR Good Practices for Performance-Based Risk-Sharing Arrangements Task Force. Value Health.

[37] Breccia M., Olimpieri P.P., Celant S., Olimpieri O., Pane F., Iurlo A., Summa V., Corradini P., Russo P. (2022). Management of chronic myeloid leukaemia patients treated with ponatinib in a real-life setting: A retrospective analysis from the monitoring registries of the Italian Medicines Agency (AIFA). Br. J. Haematol..

[38] Carlson J.J., Gries K.S., Yeung K., Sullivan S.D., Garrison L.P. (2014). Current status and trends in performance-based risk-sharing arrangements between healthcare payers and medical product manufacturers. Appl. Health Econ. Health Policy.

[39] Carlson J.J., Chen S., Garrison L.P. (2017). Performance-Based Risk-Sharing Arrangements: An Updated International Review. Pharmacoeconomics.

[40] Dabbous M., Chachoua L., Caban A., Toumi M. (2020). Managed Entry Agreements: Policy Analysis From the European Perspective. Value Health.

[41] Carlson J.J., Sullivan S.D., Garrison L.P., Neumann P.J., Veenstra D.L. (2010). Linking payment to health outcomes: A taxonomy and examination of performance-based reimbursement schemes between healthcare payers and manufacturers. Health Policy.

[42] Ferrario A., Kanavos P. (2013). Managed Entry Agreements for Pharmaceuticals: The European Experience.

[43] Setti F., Mocchi A., Cappellaro E. (2015). 11. Italy. Pricing and Reimbursement Questions.

[44] Folino-Gallo P., Montilla S., Bruzzone M., Martini N. (2008). Pricing and reimbursement of pharmaceuticals in Italy. Eur. J. Health Econ..

[45] Garattini L., Casadei G. (2011). Risk sharing agreements: What lessons from Italy?. Int. J. Technol. Assess. Health Care.

[46] Chin W.W. (2015). A Delicate Balance—Pharmaceutical Innovation and Access. N. Engl. J. Med..

[47] Brown A., Colasante W., Kremer T., Ludidi P., Misto I. (2017). Pricing and Market Access Outlook.

[48] Smith J.C., Medalia C. (2015). Health Insurance Coverage in the United States: 2014.

[49] Young R.A., de Voe J.E. (2012). Who will have health insurance in the future? An updated projection. Ann. Fam. Med..

[50] Austin D.A. (2014). Medical Debt as a Cause of Consumer Bankruptcy. Maine Law Rev..

[51] Aitken M., Valkova S. (2013). Avoidable Costs in U.S. Healthcare: The $200 Billion Opportunity from Using Medicines More Responsibly.

[52] Tunis S.R., Pearson S.D. (2006). Coverage options for promising technologies: Medicare’s “Coverage with evidence development. Health Aff..

